# What is the impact of vitamin D supplementation on glycemic control in people with type-2 diabetes: a systematic review and meta-analysis of randomized controlled trails

**DOI:** 10.1186/s12902-022-01209-x

**Published:** 2023-01-16

**Authors:** Mohammad Ashraf Farahmand, Elnaz Daneshzad, Teresa T. Fung, Fawzia Zahidi, Maryam Muhammadi, Nick Bellissimo, Leila Azadbakht

**Affiliations:** 1 Department of Community Nutrition, School of Nutritional Sciences and Dietetics, Tehran University of Medical Sciences (TUMS), PO BOX: 1416643931, Tehran, Iran; 2 Public Health Faculty, Kabul University of Medical Science (KUMS), Kabul, Afghanistan; 3 Non-Communicable Diseases Research Center, Alborz University of Medical Sciences, Karaj, Iran; 4 Department of Nutrition, Harvard TH Chan School of Public Health, Boston, USA; 5 Department of Nutrition, Simmons University, Boston, MA USA; 6 Critical Nursing Care Department, Kabul University of Medical Science (KUMS), Kabul, Afghanistan; 7 School of Nutrition, Toronto Metropolitan University, Toronto, ON Canada; 8 Diabetes Research Centre, Endocrinology and Metabolism Clinical Sciences Institute, Tehran University of Medical Sciences, Tehran, Iran

**Keywords:** Vitamin D, Supplementation, Type 2 diabetes, Glycemic control, Systematic review, Meta-analyses

## Abstract

**Background:**

There is conflicting evidence on the effect of vitamin D on glycemic control. Therefore, in the current meta-analyses, we aimed to assess the effect of vitamin D supplementation on the glycemic control of type 2 diabetes (T2D) patients.

**Methods:**

We conducted a comprehensive search in electronic databases including; PubMed/Medline, Web of Science, Scopus, Embase, Cochrane Central Register of Controlled Trials (CENTRAL), and NIH’s Clinical Trials Registry, from the inception of each database up to January first, 2021.

**Results:**

A total of 46 randomized controlled trials (RCTs) consisting of 2164 intervention subjects and 2149 placebo controls were included in this meta-analysis. Pooled analyses for HbA1c showed a significant change between the intervention and placebo group, the weighted mean difference (WMD)(95% confidence interval(CI)) was -0.20%(-0.29, -0.11) with *P* < 0.001. Analyses for assessing changes in FPG found a significant reduction in the intervention group after vitamin D supplementation, the WMD (95%CI) was -5.02 mg/dl (-6.75,-3.28) with *P* < 0.001. The result of pooled analyses for HOMA-IR revealed a significant change between the intervention and control group, the WMD (95%CI) was -0.42(-0.76, -0.07) with *P* = 0.019. The subgroup analyses showed the most efficacy in a higher dose and short intervention period and in subjects with deficient vitamin D status.

**Conclusion:**

Vitamin D supplementation might be beneficial for the reduction of FPG, HbA1c, and HOMA-IR in type 2 diabetes patients with deficient vitamin D status. This effect was especially prominent when vitamin D was given in large doses and for a short period of time albeit with substantial heterogeneity between studies and a probability of publication bias.

**Supplementary Information:**

The online version contains supplementary material available at 10.1186/s12902-022-01209-x.

## Introduction

Type 2 diabetes mellitus is a serious public health concern and the global prevalence has continued to rise over the past three decades [1, 2]. Globally, type 2 diabetes is ranked as the ninth leading cause of mortality contributing to more than 1 million deaths yearly and with an estimated 462 million cases in 2017 [2]. Diabetes mellitus is a group of metabolic disorders characterized by long-term hyperglycemia resulting from defects in insulin action, insulin secretion, or both [3]. Being diagnosed with and managing diabetes often affects an individuals quality of life and is also the leading cause of significant morbidity and premature mortality, as well as a major risk factor for adverse complications such as blindness, stroke, heart attack, amputation, and kidney failure [4, 5]. Although therapies for type 2 diabetes have improved over the last few decades, new insights for the prevention and management of type 2 diabetes remain necessary due to the increased prevalence of the disease.

Over the past decade, vitamin D has gained substantial interest for potential extra skeletal outcomes in various disease conditions, including diabetes [6, 7]. Vitamin D has been hypothesized to exhibit anti-diabetic properties by regulating insulin secretion or insulin sensitivity, producing anti-inflammatory effects, and down-regulation of elevated parathyroid hormone levels, which impair insulin secretion [8–11]. However, while there has been research suggesting that vitamin D may play an important role in glucose homeostasis, the results are conflicting. Many cross-sectional studies have found that insufficient vitamin D status was associated with the development of type 2 diabetes, diabetic complications, obesity, and metabolic syndrome [12–15]. A meta-analysis of observational studies indicated that low serum vitamin D levels were relatively associated with the prevalence of type 2 diabetes or metabolic syndrome [16]. Furthermore, prospective cohort studies have revealed inverse associations between baseline serum 25-OH-vitamin D [25(OH)D] and future risk of type 2 diabetes, diabetic complications, hyperglycemia, and insulin resistance [17–20]. In contrast, a meta-analysis of four prospective cohort studies suggested that there was no association between vitamin D intake and type 2 diabetes [21]. Evidence-based on Genetic Mendelian randomization analysis in a large sample of European ancestry did not support a causal association between total 25(OH)D or 25(OH)D metabolites and risk of type 2 diabetes [22].

Previous intervention studies evaluating the effect of vitamin D supplementation on glycemic control have produced conflicting findings [23–27]. Many previous meta-analyses of intervention studies exploring the effect of vitamin D supplementation on glycemic control had methodological limitations and the findings revealed inconsistencies [28–31]. Discrepancies in the findings and methodological limitations of individual studies in the literature, in addition to inconsistencies in the results of previous meta-analyses warrant the need for a comprehensive meta-analysis evaluating these relationships. Contrary to previous meta-analyses, only studies with vitamin D supplementation were included to isolate the potential effect of vitamin D independently, while studies with co-supplementation were excluded. Furthermore, there is a need for an updated systematic review and meta-analysis due to the increased volume of new or eligible studies in the literature evaluating the relationship between vitamin D supplementation and glycemic control since the publication of the most recent previous review.

Therefore, the objective of the current systematic review and meta-analysis of randomized controlled trials (RCTs) was to examine the effect of vitamin D supplementation on glycemic control indicators including fasting plasma glucose (FPG), HbA1c, and homeostasis model assessment insulin resistance (HOMA-IR) in patients with type 2 diabetes.

## Materials and method

The study was performed according to the Preferred Reporting Items for Systematic Review and Meta-analysis (PRISMA) statement guideline [32]. Since the present study utilized data from previously published studies, no patient consent or ethical approval was required. The protocol of this study was registered to Prospero online database (ID: 327,944).

### Data sources and searches

A comprehensive search of online electronic databases from the inception of each database until January 1^st^, 2021 was completed. The databases included PubMed/Medline, Web of Science, Scopus, Embase, and Cochrane Central Register of Controlled Trials (CENTRAL). NIH’s Clinical Trials Registry (www.clinicaltrials.gov) was searched for completed but unpublished studies. The title/abstract search strategy based on syntax and MeSH terms of the following keywords and terms was performed: [(vitamin D supplementation OR vitamin D OR vitamin D2 OR vitamin D3 OR cholecalciferol OR ergocalciferol OR alphacalcidol OR alfacalcidol OR paricalcitol OR doxercalciferol OR calcitriol OR 25-Hydroxyvitamin D OR vitamin D) AND (Diabete OR diabetes mellitus OR T2DM OR hyperglycemia OR hyperglycaemia OR glucose OR HbA1c OR glycated hemoglobin OR insulin resistance OR insulin sensitivity OR HOMA OR glucose homeostasis OR insulin secretion OR insulin OR beta cell function OR glycemic control OR glucose tolerance OR glucose metabolism OR fasting blood glucose OR FBS OR Diabetes Mellitus OR Glycated Hemoglobin A OR Insulin Resistance) AND (randomized controlled trial OR controlled clinical trial OR random OR clinical trial OR controlled trial OR RCT) NOT (review OR animal)].

In addition, we hand-searched the reference lists of the included articles and previous reviews for additional relevant studies to help prevent missing any eligible studies. There were no restrictions on the date or language of publications. All eligible studies were included and duplicated citations were removed prior to screening using the Mendeley software.

## Study selection

The inclusion criteria were: (1) RCT design, (2) adult population (age ≥ 18y), (3) population diagnosed with type 2 diabetes, (4) reported at least one glycemic control outcome (FBG or insulin or HbA1c or insulin resistance), (5) insulin resistance estimated by HOMA-IR; ((glucose, [mmol/L] × insulin [mU/L])/22.5) [33], (6) data reported as mean ± SD along with 95%CI,(7) the intervention group provided Vitamin D supplementation, while the control group provided placebo.

The exclusion criteria were: (1) letters, comments, short communications, meta-analyses, reviews, abstracts, or animal studies, (2) incomplete reporting of necessary data, (3) interventions with co-supplementation, (4) follow-up less than two months, (5) populations with type 1 diabetes, high-risk population of diabetes, pre-diabetic, gestational diabetes, post-partum diabetes, and diabetic nephropathy.

### Data extraction and risk of bias assessment

Two reviewers (M.A.F, M.M) independently screened studies based on the inclusion and exclusion criteria. First, the title and abstract of all retrieved studies were screened to identify potentially relevant articles. Studies judged to be relevant were selected for full-text review. During the full-text screening, each article was analyzed by both reviewers which determined whether the article qualified for inclusion. Any conflicting decisions between reviewers to include a given study were resolved initially by consensus, but in case of no resolution, a third author (L.A) was consulted for the final decision. Additionally, if the data was not available or unclear in the published article, the author was contacted by e-mail to request the necessary data.

We evaluated the methodological quality of each included trial using the validated 6-item Cochrane risk of the bias assessment tool, 2009 [34]. Using this tool, each study was assigned a “high”, “some concerns” or “low” score for each of the following items: random sequence generation; allocation concealment; blinding of participants and personal; blinding of outcome assessment; incomplete outcome data and other sources of bias.

Required data from each eligible study were extracted by two independent investigators. The mean and standard deviation (SD) and corresponding 95% CIs for the effect of vitamin D supplementation on glycemic control were extracted from each study. Study information (authors, year, country, sample size), participants’ characteristics (gender, age, body mass index (BMI), vitamin D status), vitamin D supplementation characteristics (treatment, type, dose, and therapy duration), and results (baseline and post-intervention serum vitamin D, FBG, HbA1c, and HOMA-IR) were extracted. Serum vitamin D status was defined as deficient (serum 25(OH)D concentrations < 50 nmol/l), insufficient (50–75 nmol/l), and sufficient (> 75 nmol /l) [35].

### Data synthesis and analysis

The STATA (version 13.0; College Station, Texas) software was used for all statistical analyses. We extracted data for continuous outcomes including the mean and SD change in FPG, HbA1c, and HOMA-IR with consideration of the 95% CI. A random-effects model (DerSimonian and Laird method) was used to estimate the pooled results. The Chi-square test and I^2^ statistic (%) were used to assess heterogeneity. The I^2^ index calculated as the proportion of total variation attributable to between-study variation was used to determine statistical heterogeneity between the studies. An I^2^ value of greater than 75% was considered substantial heterogeneity [36]. Subgroup analyses by continent, study type, BMI, vitamin D status, vitamin D dose, study duration, and supplementation type were performed using Cochran’s Q test and the I2 statistic to assess the possible sources of heterogeneity. Moreover, we assessed the publication bias using funnel plots and Egger’s regression test. Also, sensitivity analysis was performed to distinguish the extent to which summary estimates probably might be related to a particular study or group of studies. Data analyses were performed using Stata Software, version 14. *P*-values were reported as statistically significant at the < 0.05 level.

## Result

### Search results

**Fig. 1:**
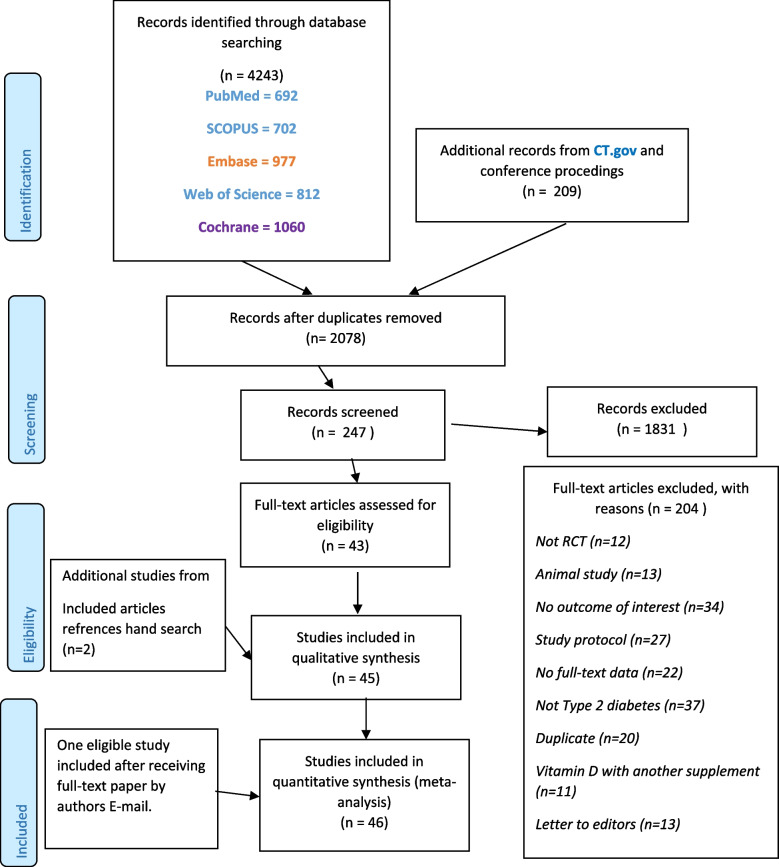
PRISMA flow diagram of search and selection of studies

We identified 4452 articles in our initial search. After excluding duplicates and those that did not meet the inclusion criteria in the title and abstract screening, we identified 247 articles for full-text screening. After the full-text review, we excluded an additional 204 studies. A total of 43 papers were included in the current meta-analysis. Moreover, two additional studies were included after hand-searching the reference lists of the included papers [37, 38], and another study was included after receiving the full text of the paper by the author's email [39]. Among the 46 included studies, 35 reported an appropriate effect size for FPG, 42 for HbA1c, and 19 for HOMA-IR. A flow diagram of the study selection is presented in Fig. 1.

### Study characteristics

Characteristics of the included studies are summarized in Table 1. 31 studies were conducted in Asia [24, 39–68], 9 in Europe [23, 27, 69–74], 3 in America [75–77], 1 in Africa [78], 1 in Australia [26] and 1 was conducted in 33 different countries [37]. A total of 4313 participants with type 2 diabetes were included in the current study, 2164 represented the intervention group and 2149 represented the control group. Across the included studies, the mean age of participants was 56.51 years and the mean BMI was 29.32. The baseline vitamin D status of 34 studies was classified as deficient, 6 studies as insufficient, 4 studies as sufficient and 2 studies did not report baseline vitamin D status. All studies included participants from both genders, except for one study with a population of only women [66]. The intervention ranged from 8 to 48 weeks in duration. Forty-two studies used oral vitamin D supplementation, while 4 studies used intramuscular (IM) vitamin D.

**Table 1:** Baseline characteristics of included studies

Author Year	Continent, Country	Sex	Int./cont	Mean Age(Year)	Mean BMI	Blinding	Baseline Vit.D Status	Vitamin D dose	Study Duration (week)	Outcomes^a^
Shaseb et al. 2016 [40]	Asia, Iran	both	55/57	54.90	27.2	Double Blind	Sufficient	300,000, Once	8	1&2
Farrokhian et al. 2017 [41]	Asia, Iran	both	3030	61.70	30.2	Double Blind	Deficient	50,000 /w	24	1&3
Kampman et al. 2014 [69]	Europe, Denmark	both	8/8	61.60	33.8	Double Blind	Deficient	5,600/d	12	1
Gulseth et al. 2017 [23]	Europe, Norway	both	33/29	55.50	31.8	Double Blind	Deficient	400,000, Once	24	1&2
Khan et al. 2018 [42]	Asia, Pakistan	both	70/70	54.80	NR	NR	Deficient	50,000/w	12	2
Ghavamzadeh et al. 2014 [43]	Asia, Iran	both	26/25	52.26	28.9	Double Blind	Deficient	400/d	14	2
Sadiya et al. 2015 [44]	Asia, UAE	both	43/39	49.00	37.9	Double Blind	Deficient	6,000/d	24	1&2
Dalan et al. 2016 [45]	Asia, Singapore	both	31/30	52.20	27.3	Double Blind	Deficient	4,000/d	16	2
Witham et al. 2010 [70]	Europe, UK	both	19/21	65.30	31.1	Double Blind	Deficient	100,000, Once	16	2&3
Witham et al. 2010 [70]	Europe, UK	both	18/21	66.70	33.3	Double Blind	Deficient	200,000, Once	16	2&3
Barchetta et al. 2016 [88]	Europe, Italy	both	29/26	57.40	29.3	Double Blind	Deficient	2,000/d	24	1&2&3
Momeni et al. 2017 [46]	Asia, Iran	both	29/28	62.60	NR	Double Blind	Deficient	50,000/w	8	1&2
Angellotti et al. 2018 [75]	America, USA	both	61/59	60.20	30.9	Double Blind	Insufficient	4,000/d	48	2
Omidian et al. 2019 [47]	Asia, Iran	both	32/34	50.50	27.4	Double Blind	Deficient	4,000/d	12	1&2
Rad et al. 2014 [89]	Asia, Iran	both	30/26	50.00	28.8	Double Blind	Deficient	4,000/d	8	1&2&3
Eftekhari et al. 2011 [49]	Asia, Iran	both	35/35	53.80	28.3	Double Blind	Sufficient	20/d	12	1&2&3
Ryu et al. 2014 [50]	Asia, Korea	both	64/65	54.50	24.4	Double Blind	Deficient	2,000/d	24	1&2&3
Agarwal et al. 2017 [51]	Asia, India	both	30/30	55.50	NR	Open labeled	Deficient	60,000/2w	12	1&2
Heshmat et al. 2012 [52]	Asia, Iran	both	21/21	56.20	27.7	Double Blind	Sufficient	300,000, Once	12	1&2&3
Soric et al. 2012 [76]	America, USA	both	19/18	55.50	NR	Single Blinding	NR	2,000/d	12	1&2
Shaheen et al. 2019 [53]	Asia, Pakistan	both	70/70	56.60	NR	NR	Deficient	50,000/w	12	2
Sugden et al. 2008 [72]	Europe, UK	both	17/17	64.90	31.7	Double Blind	Deficient	100,000, Once	8	2
Safarpour et al. 2020 [24]	Asia, Iran	both	42/43	50.10	30.9	Double Blind	Deficient	50,000/w	12	1&2&3
Upreti et al. 2018 [54]	Asia, India	both	30/30	49.10	25.1	Double Blind	Insufficient	60,000/w	24	1&2
Dadrass et al. 2019 [55]	Asia, Iran	Male	12/12	53.86	28.1	Double Blind	Deficient	50,000/2w	12	1&2&3
Baziar et al. 2014 [25]	Asia, Iran	both	41/40	51.30	27.3	Double Blind	Deficient	50,000/w	8	1&3
Krul-Poel et al. 2015 [27]	Europe, Netherland	both	129/132	67.00	28.6	Double Blind	Insufficient	50,000/Month	24	1&2&3
Jehle et al. 2014 [73]	Europe, Switzerland	both	29/26	66.80	28.5	Double Blind	Deficient	300,000/double	24	2&3
Nasri et al. 2014 [57]	Asia, Iran	both	30/30	55.00	NR	Double Blind	Sufficient	50,000/w	12	2
Breslavsky et al. 2013 [58]	Asia, Israel	both	24/23	66.30	29.3	Double Blind	Deficient	1,000/d	12	1&2&3
El Hajj et al. 2020 [59]	Asia, Lebanon	both	45/43	66.90	22.7	Double Blind	Deficient	30,000/w	24	1&2&3
Mirzavandi et al. 2020 [60]	Asia, Iran	both	25/25	29.90	29.9	Double Blind	Deficient	200,000/double	8	1
Imanparasta et al. 2020 [61]	Asia, Iran	both	23/23	53.20	28.3	Double Blind	Insufficient	50,000/w	16	1&2&3
Elkassaby et al. 2014 [26]	Australia, Australia	both	26/24	52.00	30.9	Double Blind	Insufficient	6,000/d	24	1&2&3
Razzaghi et al. 2017 [62]	Asia, Iran	both	30/30	59.10	26.1	Double Blind	Deficient	50,000/2w	12	1&2
Parekh et al. 2010 [63]	Asia, India	both	14/13	43.72	23.6	Double Blind	Deficient	300,000, Once	4	1&2
Al-Sofiani et al. 2015 [64]	Asia, Saudi	both	10/10	54.80	31.2	Double Blind	Deficient	5,000/d	12	1&2&3
Anyanwu et al. 2017 [78]	Africa, Nigeria	both	17/16	51.80	NR	Single Blinding	Deficient	3,000/d	12	1&2
Lo et al. 2019 [77]	America, USA	both	14/16	58.10	38.6	Double Blind	Deficient	50,000/w	24	2
Muley et al. 2019 [65]	Asia, India	both	40/30	54.00	29.2	NR	Deficient	60,000/w	8	2
Jorde et al. 2009 [74]	Europe, Norway	both	16/16	56.25	32.1	NR	Insufficient	40,000/w	24	1&2
Punthakee et al. 2012 [37]	33 country	both	607/614	67.00	30.7	Double Blind	NR	1,000/d	18	1&2
Kim et al. 2014 [66]	Asia, Korea	Female	11/13	72.20	23.9	Double Blind	Deficient	1,200/d	12	1&3
Almoushawah et al 2014 [90]	Asia, Saudi	both	91/92	54.70	31.7	Double Blind	Deficient	45,000/w	12	1&2
Bhosle et al. 2018 [68]	Asia, India	both	60/60	NR	NR	Double Blind	Deficient	60,000/w	24	1&2
Shahriari et al. 2018 [39]	Asia, Iran	both	28/29	58.50	28.0	Double Blind	Deficient	50,000/w	8	1&2

*Abbreviations* (*Int* Intervention, *Cont* Control, *BMI* Body mass index, *Vit.D* Vitamin D, *w* Week, *d* Day, NR; not reported)

^a^ *Outcomes(1* = *FPG, 2* = *HbA1c, 3* = *HOMA-IR)*

### Quality assessment

The risk of bias assessment was conducted using the Cochrane risk of bias assessment tool [34] (Supplementary figure S1). Overall, 8 out of 46 included studies were considered as ‘high quality’ due to not being classified as high risk or unclear risk of bias on any of the assessment items. Among the remaining studies, 16 were considered as ‘moderate quality’ due to being classified as unclear risk of bias on ≤ 2 items, but not having any assessment items classified as high-risk. The remaining 22 studies were considered as ‘poor quality’ due to being classified as high-risk on any of the items or having > 2 items classified as an unclear risk of bias on the six domains. Allocation concealment, blinding of participants and personal and random sequence generation were the three most common sources of bias.

### Meta-analysis

#### Vitamin D supplementation and FPG

**Fig. 2:**
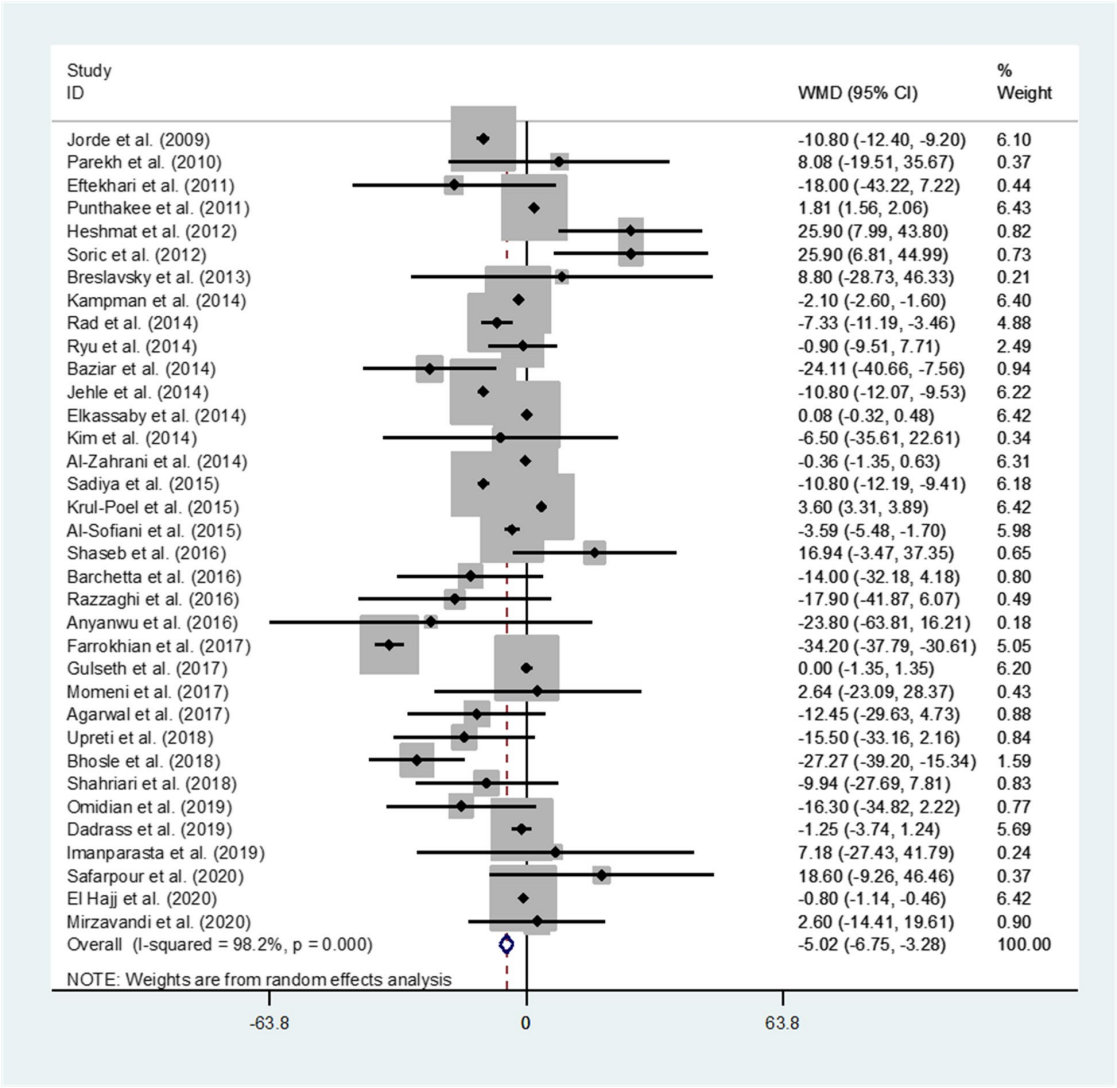
Forest plots of meta-analysis on the effect of vitamin D supplementation on FPG. Data are pooled WMDs with 95% CIs. FPG: fasting plasma glucose; WMD: weighted mean difference, CI: confidence interval. FPG is measured based on mg/dl

Thirty-five studies representing a total of 3528 participants evaluated the effect of vitamin D supplementation on FPG. In the pooled analysis for FPG, a statistically significant difference between the intervention group and placebo group (WMD: -5.02 mg/dl (-0.28 mmol/L); 95%CI: -6.75 to -3.28 (-0.37 to -0.18 mmol/L), *P* < 0.00) was observed indicating an inverse effect of vitamin D on FBG (Fig. 2). However, there was evidence of significant heterogeneity between studies (I^2^ = 98.2%; *P* < 0.001).

Subgroup analyses revealed that continent, study type, vitamin D status, BMI, supplementation type, vitamin D dosage, and study duration explained the between-study heterogeneity. These analyses revealed a significant inverse effect of vitamin D supplementation on FPG in the majority of the study subgroups. The highest magnitude of differences were observed in subjects with deficient vitamin D status (WMD (95%CI): -7.37 mg/dl (-9.82, -4.92); *P* < 0.001) (Supplementary Table S1).

Based on the findings from the meta-regression, no effect of age on the effect sizes was found (regression coefficient = 0.04; 95%CI: -0.45, 0.55; *P* = 0.856).

#### Vitamin D supplementation and HbA1c

**Fig. 3:**
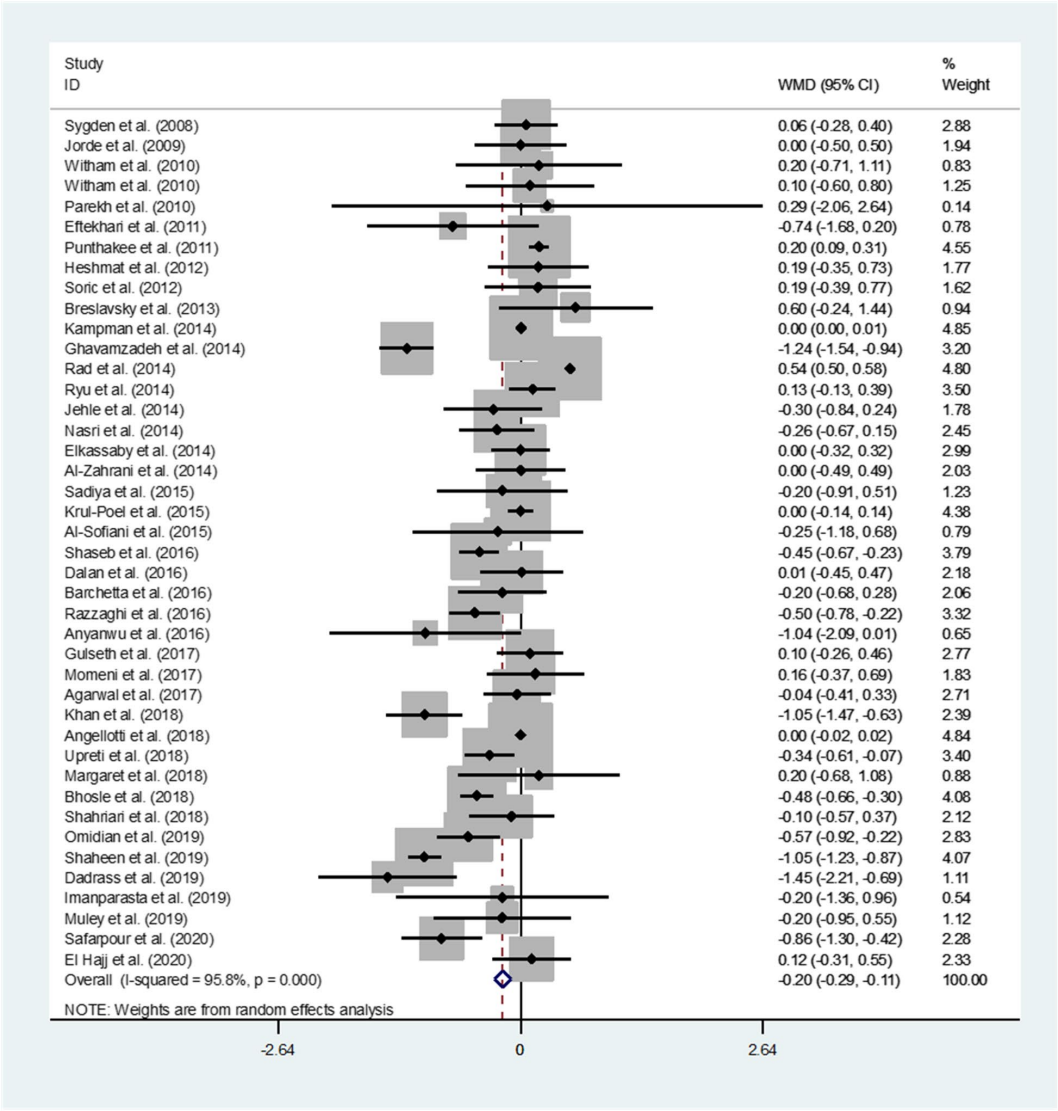
Forest plots of meta-analysis on the effect of vitamin D supplementation on HbA1c. Data are pooled WMDs with 95% CIs. HbA1c: hemoglobin A1c; WMD: weighted mean difference, CI: confidence interval. HbA1c is measured based on percentages

Forty-two studies representing a total of 4098 participants evaluated the effect of vitamin D supplementation on HbA1c. In the pooled analyses for HbA1c, a statistically significant difference between the intervention and control group (WMD (95%CI): -0.20% (-0.29, -0.11); *P* < 0.001) was observed indicating an inverse effect of vitamin D on HbA1c (Fig. 3). However, there was evidence of significant heterogeneity between studies (I^2^ = 98.2%; *p* < 0.001). In the subgroup analyses, we found that HbA1c was significantly lower in many of the subgroups including studies study populations from Asia, double-blind study designs, deficient serum vitamin D status, oral supplementation type, duration of ≤ 12 weeks, and a vitamin D dosage of > 2000 IU (Supplementary Table S2).

Based on the findings from the meta-regression, no effect of age on the effect sizes was found (regression coefficient = 0.005; 95%CI: -0.03, 0.05; *P* = 0.797).

#### Vitamin D supplementation and HOMA-IR

**Fig. 4:**
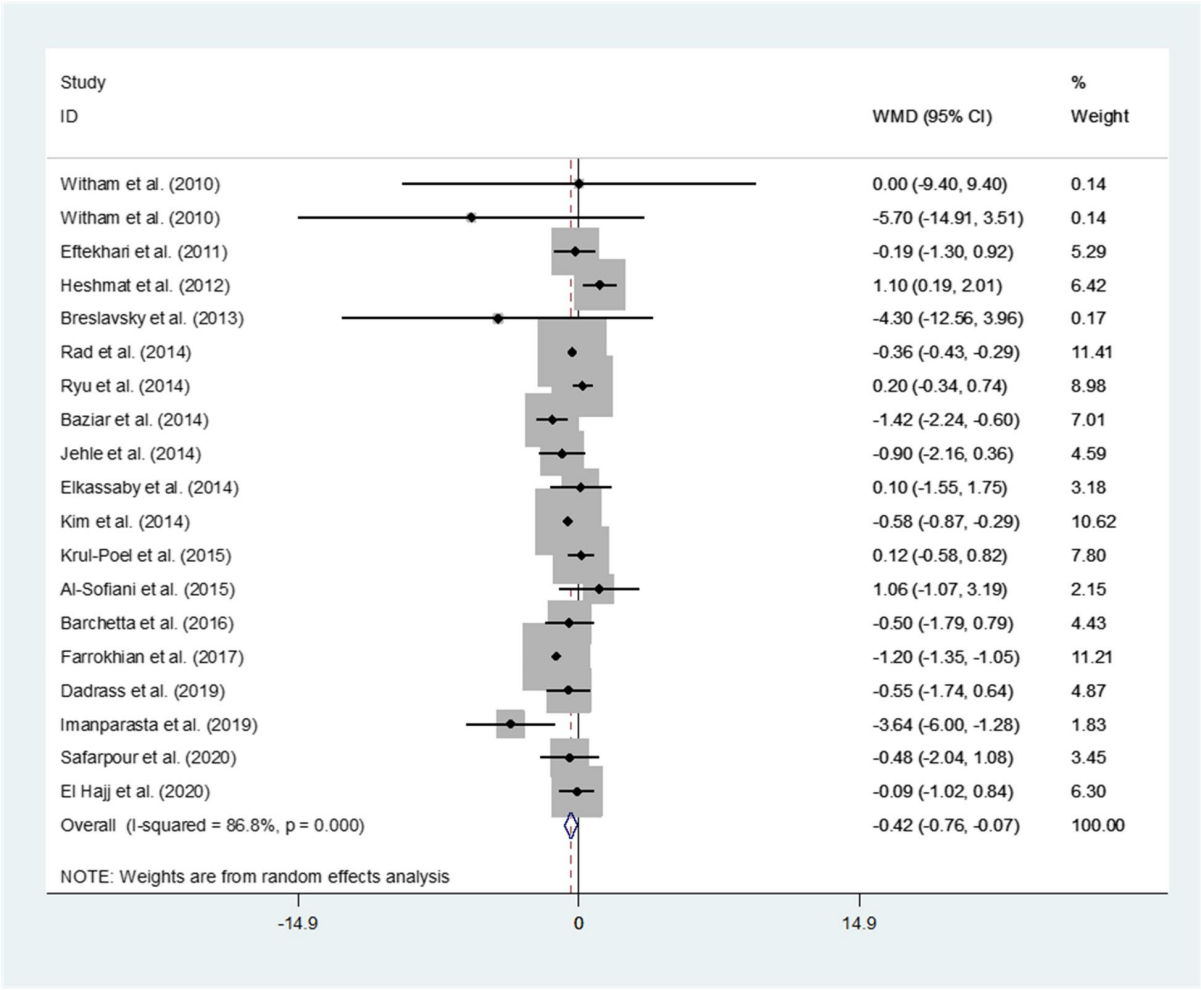
Forest plots of meta-analysis on the effect of vitamin D supplementation on HOMA-IR. Data are pooled WMDs with 95% CIs. HOMA-IR: homeostasis model assessment insulin resistance; WMD: weighted mean difference, CI: confidence interval

Nineteen studies representing a total of 1272 participants evaluated the effect of vitamin D supplementation on HOMA-IR level. In the pooled analyses for HOMA-IR, a statistically significant difference between the intervention and control group (WMD (95%CI): -0.42 (-0.76, -0.07); *P* = 0.019) was observed indicating an inverse effect of vitamin D on HOMA-IR (Fig. 4). Subgroup analyses revealed that HOMA-IR level decreased significantly in many subgroups including study populations from Asia, deficient serum vitamin D status, oral supplementation type, duration of ≤ 12 weeks, and vitamin D dose of > 2000 IU (Supplementary Table S3).

Based on the findings from the meta-regression, no effect of age on the effect sizes was found (regression coefficient = -0.006; 95%CI: -0.05, 0.03; *P* = 0.761).

### Publication bias

The funnel plot for FPG appeared to be symmetric; but the Eggers test indicated that there was probability of publication bias (Supplementary Figure S2, Egger test = 0.024). The funnel plot for HbA1c appeared to be asymmetric (Supplementary Figure S3), but the Eggers test indicated that there was weak probability of publication bias (*P* = 0.402).

Similarly, there was no evidence of publication bias with HOMA-IR based on the results of the Eggers test (*P* = 0.842). However, the funnel plot for HOMA-IR appeared to be slightly asymmetric with a few studies falling beyond the funnel, which is consistent with the substantial heterogeneity observed among studies reporting HOMA-IR (Supplementary Figure S4).

### Sensitivity analyses

In the sensitivity analyses, we did not find evidence that any particular study was the main source of heterogeneity.

## Discussion

In the current systematic review and meta-analysis of RCTs, we found that vitamin D supplementation interventions resulted in a reduction in FPG, HbA1c, and HOMA-IR for individuals with type 2 diabetes. However, there was evidence of substantial heterogeneity. The heterogeneity appeared to be partially explained by diversity between study populations for serum vitamin D status, supplementation characteristics (dose and duration), BMIs, and ethnicity.

The FPG is a commonly used measure for the detection of short-term blood glucose [79]. HbA1c is a common measure of long-term blood glucose detection [79, 80]. In the current meta-analyses, we observed a statistically significant reduction of 5.02 mg/dl in FPG and a 0.20% reduction in HbA1c levels. However, it is important to consider that a reduction of < 0.50% in HbA1c is not considered clinically significant [81]. Findings suggest that high doses of vitamin D supplementation produced greater positive effects in vitamin D deficient, obese and Asian populations.

The baseline vitamin D status of participants was an important factor contributing to observed glycemic control outcomes. We found significant reductions in FBG and HbA1c in patients with vitamin D deficiency, but not in patients with vitamin D insufficiency or sufficiency. Similarly, even though we found that 25(OH)D increased significantly in all subgroups receiving the intervention, the greatest improvement was observed in vitamin D deficient patients. This may be due to the excess vitamin D from supplementation being stored in adipose tissue in individuals with sufficient vitamin D status rather than producing a further increase in serum level. Another reasonable explanation may be the impact of large doses of vitamin D supplementation. We found that high doses increased the likelihood of correcting vitamin D deficiency or achieving favorable levels of serum 25(OH)D, confirmed by relatively higher 25(OH)D levels in the high dose subgroup (> 2000 IU) after receiving the intervention compared to the lower dose (≤ 2000 IU). In line with our findings, a meta-analysis by Chunhua Wu et al. found that vitamin D supplementation was associated with reduced FPG and HbA1c among patients with 25-hydroxyvitamin D (25(OH) D) deficiency at baseline [29].

The dosage and duration of vitamin D supplementation are other important contributors to glycemic control outcomes for patients with type 2 diabetes. High doses of vitamin D were found to improve glycemic control outcomes and was observed to be more efficient for correcting serum 25-hydroxyvitamin D level for subjects with vitamin D deficiency. The effect of the duration of vitamin D supplementation appeared to be more ambiguous amongst glycemic control outcomes. For example, we found a statistically significant reduction in FPG with both short-term (≤ 12 weeks) and long-term (> 12 weeks) vitamin D supplementation, however, the magnitude of reduction was greater with long-term (WMD: -6.74) than short-term (WMD: -2.37) supplementation. Comparatively, there was only evidence of a reduction in HbA1c and HOMA-IR outcomes with short-term (≤ 12 weeks) vitamin D supplementation.

Among different continents of study origin, vitamin D supplementation produced varying effects. The greatest reductions were observed among studies conducted in Asia and Europe, while studies conducted on other continents revealed no evidence of a difference. However, studies conducted in Asia represented the majority of the included RCTs for the meta-analysis and it is possible that this analysis had a greater power to detect a statistically significant difference compared to other continents that may not have had a sufficient number of studies. Therefore, insufficient evidence to differentiate between the continents of study origin should be considered when interpreting the findings from the subgroup analysis. Wang et al. explained the heterogeneous responses between Asians and the other groups by demonstrating that vitamin D-binding protein polymorphism was associated with increased susceptibility to T2D in Asians, but not in Caucasians [82]. Furthermore, it is relatively understood that typically between 50 to 90% of vitamin D in the body is due to the production in the skin and the remainder is from the diet. Previous studies have found that populations with darker skin color and cultural preferences toward less exposure to the sun, which matched the profile of Asians and especially Middle Easterners, were at a higher risk of vitamin D deficiency, which in turn, was associated with greater positive effects from supplementation [83, 84]. However, there were a limited number of studies included in the analysis that represented data from America, Africa, and Australia. Therefore, future investigations on the effect of vitamin D supplementation in these continents and populations are warranted.

HOMA-IR as proposed by Matthews et al. [33], is the most frequently employed technique as the index of insulin resistance (IR) and has proved to be a robust tool for the assessment of IR [85, 86]. The concept of IR is generally defined as the reduced biological action of insulin, such as inhibition of hepatic glucose production and insulin-mediated glucose disposal [87]. In the current meta-analysis, a significant reduction in HOMA-IR was found in the vitamin D intervention compared to the control group with the lowest between-study heterogeneity found for studies with a short-duration intervention (≤ 12 weeks) in the subgroup analysis. Similar to our findings, Hu Z et al. revealed that decreases in HOMA-IR and changes in insulin resulting from a vitamin D supplementation intervention were only observed in short-term follow-up studies and not in long-term studies [30]. It was hypothesized that this may be due to higher doses of vitamin D being provided in short-term interventions in comparison to doses provided in longer-term studies. Our findings are comparable to results from another meta-analysis by Xinyi Li et al. revealing an improvement in HOMA-IR for subjects in the vitamin D intervention group and subjects with baseline vitamin D deficiency [28].

We conducted a comprehensive systematic review and meta-analysis on a large number of RCTs that investigated the effect of vitamin D supplementation on glycemic control outcomes including FPG, HbA1c, and HOMA-IR. Part of the objective for this meta-analysis was to overcome methodological concerns present in previous meta-analyses, to provide updated findings, and transition from previous inconsistencies toward more conclusive results. However, potential limitations should be considered when interpreting our findings. Although we conducted a comprehensive search, appropriate publications being missed remains a possibility. The relatively short intervention durations, small sample sizes, and variations in intervention doses and durations of some studies, in addition to the moderate to substantial heterogeneity observed in some of our results, may limit our ability to determine the effects of vitamin D supplementation and draw meaningful conclusions. Finally, some of the included RCTs did not consider the effect of sun exposure, diet, and physical activity on the findings which may have directly influenced vitamin D status, or did not consider the consumption of antidiabetic medications that may have masked the benefits of vitamin D supplementation. Another factor that may pose a challenge to interpreting the study results related to many studies which did not provide enough details to allow accurate qualitative assessment of how each study handled randomization, allocation, blinding, and missing data.

## Conclusion

The present meta-analysis found that vitamin D supplementation may be beneficial for the reduction of FPG, HbA1c, and HOMA-IR in patients with type 2 diabetes and deficient vitamin D status. This effect was especially prominent when vitamin D was given in large doses and for a short period of time albeit with substantial heterogeneity between studies and a probability of publication bias. In conclusion, this study is in agreement with previous findings on the potential of vitamin D supplementation along with other antidiabetic drugs to improve glycemic control and prevent diabetic complications.

## Supplementary Information

**Additional file 1:** **Supplementary Figure S1.** Risk  of  bias  assessment  for included  studies  using  the  Cochrane  Collaboration  tool  across six  domains. **Supplementary Table S1.** Summary table of subgroup analyses; FPG changes. **Supplementary Table S2.** Summary table of subgroup analyses; HbA1c changes. **Supplementary Table S3.**  Summary table of subgroup analyses; HOMA-IR changes. **Supplementary Figure S2.** Funnel plot for fasting plasma glucose. **Supplementary Figure S3.** Funnel plot for HbA1c. **Supplementary Figure S4.** Funnel plot for HOMA-IR.

## Data Availability

The datasets used and/or analyzed in the current study are available from the corresponding author upon reasonable request.

## Abbreviations

T2D: Type 2 diabetes
FPG: Fasting plasma glucose
HbA1c: Hemoglobin A1c
HOMA-IR: Homeostasis model assessment – insulin resistance
RCT: Randomized controlled trials
CI: Confidence interval
WMD: Weighted mean difference
