# Supplementary material for: What is the impact of vitamin D supplementation on glycemic control in people with type-2 diabetes: a systematic review and meta-analysis of randomized controlled trails
Source: BMC Endocr Disord. 2023 Jan 16;23:15. doi: 10.1186/s12902-022-01209-x (PMC9841647; doi:10.1186/s12902-022-01209-x)
Supplement: Supplementary file 1 — Additional file 1: Supplementary Figure S1. Risk of bias assessment for included studies using the Cochrane Collaboration tool across six domains. Supplementary Table S1. Summary table of subgroup analyses; FPG changes. Supplementary Table S2. Summary table of subgroup analyses; HbA1c changes. Supplementary Table S3. Summary table of subgroup analyses; HOMA-IR changes. Supplementary Figure S2. Funnel plot for fasting plasma glucose. Supplementary Figure S3. Funnel plot for HbA1c. Supplementary Figure S4. Funnel plot for HOMA-IR. [file 12902_2022_1209_MOESM1_ESM.docx]

|  |  | |
| --- | --- | --- |
| Domains:  D1: Random Sequence Generation  D2: Allocation Concealment  D3: Blinding of Participants and Personal  D4: Blinding of Outcome Assessment  D5: Incomplete Outcome Data  D6: Other Bias | Judgement  High  Some Concerns  Low | 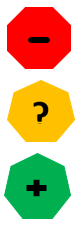 |
| **Supplementary Figure S1.** Risk of bias assessment for included studies using the Cochrane Collaboration tool across six domains. | | |

| **Supplementary Table S1.** Summary table of subgroup analyses; FPG changes. | | | | | | |
| --- | --- | --- | --- | --- | --- | --- |
| ***Sub group*** | ***Potential factor*** | ***Number of studies*** | ***WMD ^*^ (95%CI)*** | ***I^2^ (%)*** | ***P* ^ᵻ^**  ***Heterogeneity*** | ***P* ^ᵻ^**  ***Within*** |
| ***Continent*** | Asia | 25 | -7.09(-10.62, -3.56) | 95.8 | <0.01 | <0.001 |
|  | Europe | 6 | -4.49(-9.25, 0.26) | 99.5 | <0.01 | 0.064 |
|  | America | 1 | 25.9(6.81, 0.26) | - | - | 0.008 |
|  | Africa | 1 | 1.81(1.56, 2.06) | - | - | 0.244 |
|  | Australia | 1 | 0.08(-0.32, 0.48) | - | - | 0.693 |
| ***Study type*** | Double blind | 31 | -4.72(-6.45, -2.99) | 98.2 | <0.01 | <0.001 |
|  | Single blind | 2 | 4.29(-44.00, 52.58) | 79.3 | 0.028 | 0.862 |
|  | Open labeled | 1 | -12.45(-29.63, 4.73) | - | - | 0.155 |
| ***Vitamin D*** | Sufficient | 4 | 9.64(-14.52, 33.80) | 74.8 | 0.019 | 0.434 |
| ***Status*** | Insufficient | 5 | -2.72(-6.76, 1.32) | 99.1 | <0.01 | 0.187 |
|  | Deficient | 25 | -7.37(-9.82, -4.91) | 96.8 | <0.01 | <0.001 |
| ***BMI*** | Normal weight | 4 | -0.80(-1.14, -0.46) | 0.00 | 0.909 | <0.001 |
|  | Over weight | 16 | -4.84(-10.79, 1.10) | 97.2 | <0.01 | 0.110 |
|  | Obese | 10 | -5.90(-8.43, -3.37) | 99.1 | <0.01 | <0.001 |
| ***Supplementation*** | IM | 4 | 9.00(-13.04, 31.03) | 87.9 | <0.01 | 0.424 |
| ***Type*** | Oral | 31 | -4.98(-6.76, -3.29) | 98.1 | <0.01 | <0.001 |
| ***Vitamin D*** | >2000 IU | 27 | -6.56(-8.63, -4.49) | 97.4 | <0.01 | <0.001 |
| ***Dose*** | <=2000 IU | 8 | 2.49(0.73, 4.24) | 92.7 | <0.01 | 0.006 |
| ***Study duration*** | <= 12 week | 20 | -2.37(-4.27, -0.47) | 67.1 | <0.01 | 0.014 |
|  | >12 week | 14 | -6.74(-9.02, -4.46) | 99.2 | <0.01 | <0.001 |
| **All study combination** | **-** | **35** | **-5.02(-6.75, -3.28)** | **98.2** | **0.00** | **<0.001** |
| ^*^Weighted mean difference,  **^ᵻ^**P-value, IM=intramuscular, IU= international units,  FPG = fasting plasma glucose. | | | | | | |

| **Supplementary Table S2.** Summary table of subgroup analyses; HbA1c changes. | | | | | | |
| --- | --- | --- | --- | --- | --- | --- |
| ***Sub group*** | ***Potential factor*** | ***Number of studies*** | ***WMD ^a^ (95%CI)*** | ***I^2^ (%)*** | ***P* ^ᵻ^**  ***Heterogeneity*** | ***P* ^ᵻ^**  ***Within*** |
| ***Continent*** | Asia | 27 | -0.31(-0.61, -0.01) | 96.6 | <0.01 | 0.040 |
|  | Europe | 9 | 0.00(0.00, 0.01) | 0.00 | 0.96 | <0.001 |
|  | America | 3 | 0.00(-0.02, 0.02) | 0.00 | 0.737 | 0.970 |
|  | Africa | 1 | -1.04(-2.09, 0.01) | - | - | 0.052 |
|  | Australia | 1 | 0.00(-0.32, 0.32) | - | - | 1.000 |
| ***Study type*** | Double blind | 35 | -0.14(-0.23, -0.04) | 95.8 | <0.01 | 0.004 |
|  | Single blind | 2 | -0.34(-1.54, -0.11) | 75.3 | 0.044 | 0.572 |
|  | Open labeled | 1 | -0.04(-0.41, 0.33) | - | - | 0.831 |
| ***Vitamin D*** | Sufficient | 4 | -0.30(-0.59, 0.00) | 44.8 | 0.143 | 0.051 |
| ***Status*** | Insufficient | 6 | -0.02(-0.10, 0.05) | 19.6 | 0.286 | 0.527 |
|  | Deficient | 30 | -0.25(-0.42, -0.08) | 96.9 | <0.01 | 0.004 |
| ***BMI*** | Normal weight | 3 | 0.13(-0.09, 0.35) | 0.00 | 0.990 | 0.248 |
|  | Over weight | 17 | -0.29(-0.61, 0.03) | 96.0 | <0.01 | 0.081 |
|  | Obese | 14 | 0.02(-0.02, 0.06) | 55.9 | 0.006 | 0.369 |
| ***Supplementation*** | IM | 4 | -0.25(-0.58, 0.08) | 38.8 | 0.179 | 0.139 |
| ***Type*** | Oral | 38 | -0.19(-0.29, -0.10) | 96.1 | <0.01 | <0.001 |
| ***Vitamin D*** | >2000 IU | 31 | -0.23(-0.34, -0.13) | 96.6 | <0.01 | <0.001 |
| ***Dose*** | <=2000 IU | 11 | -0.08(-0.36, 0.19) | 88.5 | <0.01 | 0.564 |
| ***Study duration*** | <= 12 week | 23 | -0.28(-0.48, -0.09) | 97.4 | <0.01 | 0.004 |
|  | >12 week | 19 | -0.12(-0.26, 0.02) | 84.8 | <0.01 | 0.085 |
| **All study combination** | **-** | **42** | **-0.20(-0.29, -0.11)** | **95.8** | **<0.01** | **<0.001** |
| ^*^Weighted mean difference, **^ᵻ^**P-value, IM=intramuscular, IU= international units,  HbA1c = hemoglobin A1c. | | | | | | |

| **Supplementary Table S3.**  Summary table of subgroup analyses; HOMA-IR changes. | | | | | | |
| --- | --- | --- | --- | --- | --- | --- |
| ***Sub group*** | ***Potential factor*** | ***Number of studies*** | ***WMD ^a^ (95%CI)*** | ***I^2^ (%)*** | ***P* ^ᵻ^**  ***Heterogeneity*** | ***P* ^ᵻ^**  ***Within*** |
| ***Continent*** | Asia | 13 | -0.45(-0.85, -0.05) | 90.8 | <0.01 | 0.027 |
|  | Europe | 5 | -0.21(-0.76, 0.34) | 0.00 | 0.468 | 0.453 |
|  | Australia | 1 | 0.10(-1.55, 1.75) | - | - | 0.906 |
| ***Vitamin D*** | Sufficient | 2 | 0.50(-0.77, 1.76) | 67.9 | 0.078 | 0.442 |
| ***Status*** | Insufficient | 3 | -0.86(-2.71, 1.00) | 78.0 | 0.011 | 0.366 |
|  | Deficient | 14 | -0.56(-0.95, -0.18) | 88.6 | <0.01 | 0.004 |
| ***BMI*** | Normal weight | 3 | -0.21(-0.78, 0.36) | 70.0 | 0.036 | 0.479 |
|  | Over weight | 10 | -0.45(-0.94, 0.05) | 67.2 | 0.001 | 0.078 |
|  | Obese | 6 | -0.56(-1.46, 0.33) | 40.1 | 0.138 | 0.219 |
| ***Supplementation*** | IM | 2 | 0.15(-1.81, 2.11) | 84.4 | 0.011 | 0.882 |
| ***Type*** | Oral | 17 | -0.5(-0.86, -0.14) | 87.1 | <0.01 | 0.006 |
| ***Vitamin D*** | >2000 IU | 11 | -0.53(-1.01, -0.05) | 91.9 | <0.01 | 0.031 |
| ***Dose*** | <=2000 IU | 8 | -0.22(-0.62, 0.17) | 32.2 | 0.163 | 0.269 |
| ***Study duration*** | <= 12 week | 9 | -0.37(-0.73, -0.01) | 62.4 | 0.006 | 0.045 |
|  | >12 week | 10 | -0.56(-1.23, 0.12) | 81.0 | <0.01 | 0.106 |
| ***All study combination*** | **-** | **19** | **-0.42(-0.76, -0.07)** | **86.8** | **0.00** | **-** |
| ^*^Weighted mean difference, **^ᵻ^**P-value, IM=intramuscular, IU= international units,  HOMA-IR= Homeostasis model assessment for insulin resistance. | | | | | | |

| 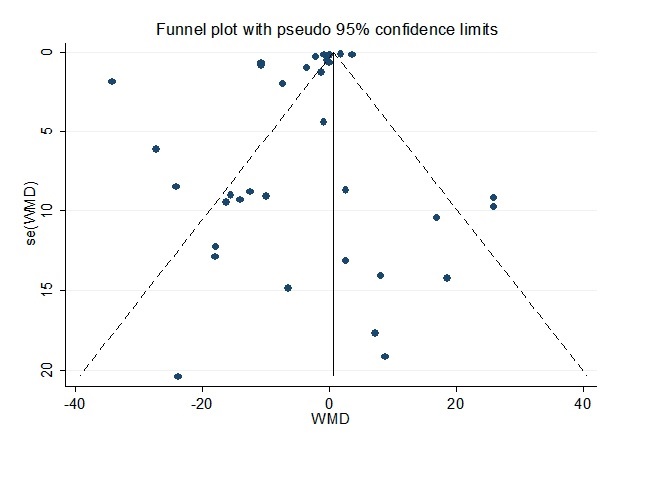 |
| --- |
| **Supplementary Figure S2.** Funnel plot for fasting plasma glucose |

| 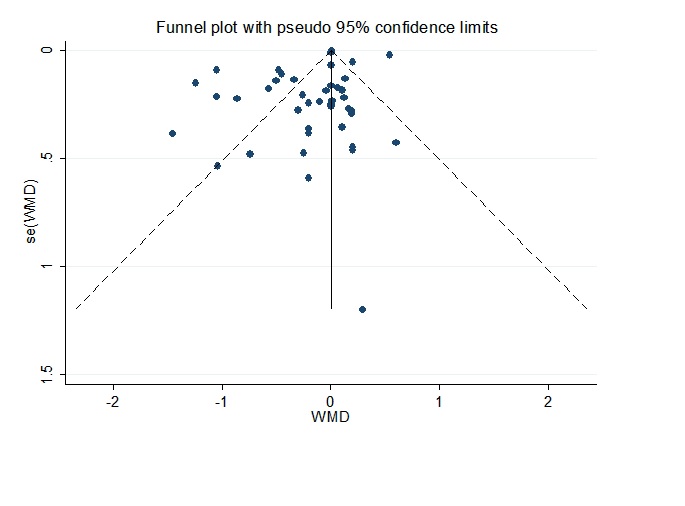  **Supplementary Figure S3.** Funnel plot for HbA1c |
| --- |

| 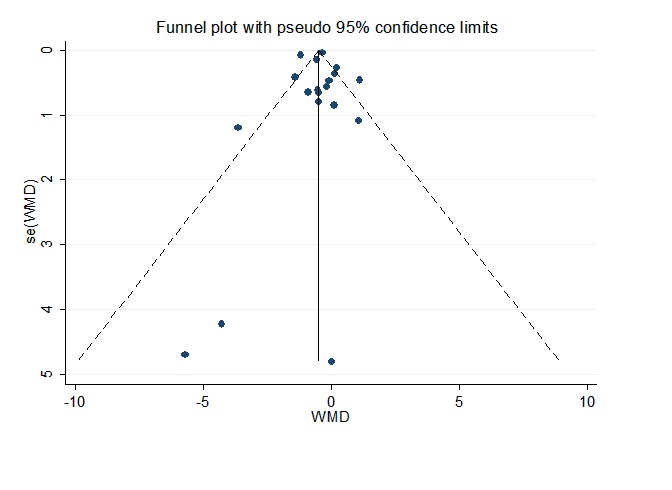 |
| --- |
| **Supplementary Figure S4.** Funnel plot for HOMA-IR |
